# State Children and the War

**Published:** 1914-11-14

**Authors:** Albert Spicer, Louise Oliver, Franca Buxton, Henrietta O. Barnett

**Affiliations:** Chairman.; Vice-Chairman.; Hon. Treasurers.; Hon. Treasurers.; Hon. Secretary. 53 Victoria Street, S.W.


### THE EDITOR'S LETTER-BOX.

### State Children and the War.

*To the Editor of* The Hospital.

Sir,—That children are a nation's greatest asset is a truth which is being slowly realised by the English people. But there are times when the slow realisation of a truth suddenly changes to conviction, and the present is such a time.

The L.G.B. has recently and most opportunely issued a circular on the subject of out-relief to widows and children, which points out that "the value of true home life in the education and training of the child is admittedly so great that, wherever the elements of this are already in existence, Guardians would do well to offer it every encouragement, and to take such action in regard to the granting of relief as will least interfere with the unity of the family."

Good homes with good mothers could thus be preserved, second rate homes be raised to a better level by friendly influence, while children, removed from homes that are unfit, should be brought up in surroundings as much like those of the ordinary family as possible.

It is only by the adoption of simple, natural home-like life and training that we shall secure to each child the best preparation for its work in the world, and it is to those methods which allow of the highest development, and not to big institutions and numbing routine, that we must turn for dealing with the increased number of children who will become dependent on the Poor Law.—We are, Sir, your obedient servants,

(Signed) Lytton, Chairman.
Albert Spicer, Vice-Chairman.
Louise Oliver, Franca Buxton, } Hon. Treasurers.
Henrietta O. Barnett, Hon. Secretary.

53 Victoria Street, S.W.
November 9, 1914.

